# The Brazilian Portuguese version of the Exercise Adherence Rating Scale (EARS-Br) showed acceptable reliability, validity and responsiveness in chronic low back pain

**DOI:** 10.1186/s12891-020-03308-z

**Published:** 2020-05-12

**Authors:** Mariana Romano de Lira, Anamaria Siriani de Oliveira, Roberta Aniceto França, Ana Claudia Pereira, Emma L. Godfrey, Thais Cristina Chaves

**Affiliations:** 1 Post Graduate Program on Rehabilitation and Functional Performance, Ribeirão Preto Medical School, University of São Paulo, Ribeirão Preto, São Paulo, Brazil; 2 Department of Health Sciences and Post Graduate Program on Rehabilitation and Functional Performance, Ribeirão Preto Medical School, University of São Paulo, Ribeirão Preto, São Paulo, Brazil; 3 Physical Therapy undergraduate course from Ribeirão Preto Medical School, University of São Paulo, Ribeirão Preto, São Paulo, Brazil; 4 Department of Population Health Sciences, Faculty of Life Sciences & Medicine, King’s College London, 2nd Floor Addison House, Guy’s Campus, London, SE1 1UL UK; 5 Department of Psychology, IoPPN, King’s College London, 5th Floor Bermondsey Wing, Guy’s Campus, London, SE1 9RT UK

**Keywords:** Validity studies, Chronic low Back pain, Adherence, Prescribed exercise, Responsiveness

## Abstract

**Background:**

This study aimed to adapt the Exercise Adherence Rating Scale (EARS) into Brazilian Portuguese and evaluate its measurement properties, given as reliability, validity, and responsiveness in patients with non-specific Chronic Low Back Pain (CLBP).

**Methods:**

A total of 108 patients with a mean age of 46.62 years (SD = 9.98) and CLBP participated in this longitudinal study. Participants were oriented on undertaking the prescribed exercises in the first session, and adherence behavior was assessed after 1 week, and finally reassessed after 2 weeks (test-retest reliability). Three weeks after the first assessment, they were invited again to full fill the EARS (responsiveness). The intraclass correlation coefficient (ICC_2,1_) and Cronbach’s α were used to assess test-retest reliability and internal consistency, respectively. Spearman’s correlation and confirmatory factor analysis (CFA) were used to assess construct validity, and the Receiver operating characteristic curve and area under the curve (AUC) were used to analyze responsiveness.

**Results:**

The one-factor EARS-Br (adherence behavior) structure with 6 items showed acceptable fit indexes (comparative fit index and goodness of fit index> 0.90 and root-mean-square error of approximation< 0.08). The EARS-Br scale showed acceptable internal consistency (α = 0.88) and excellent reliability (ICC = 0.91 [95% CI 0.86–0.94]). Mild to moderate correlations were observed between EARS-Br total score vs. disability, pain catastrophizing, depression/anxiety, fear-avoidance and pain intensity. A Minimally Important Change (MIC) of 5.5 in the EARS-Br total score was considered as a meaningful change in the adherence behavior (AUC = 0.82). Moderate accuracy (AUC = 0.89) was obtained for a 17/24 total EARS cutoff score after home exercise was prescribed. The sensitivity and specificity were also acceptable (greater than 80%).

**Conclusion:**

Our results demonstrated acceptable EARS-Br reliability, validity, and responsiveness for patients with CLBP. A final score of 17/24 on EARS after the prescription of home-exercise could be used as a cut-off for an acceptable adherence behavior associated with improvement in patient outcomes.

## Background

Adherence has been defined as the extent to which a person’s behavior corresponds with an agreed recommendation from a health care provider [1]. It is a multidimensional construct that can be affected by factors related to the health condition, the subject (such as self-efficacy, attitudes, psychosocial factors and socioeconomic status), and the interaction between the subject and healthcare professionals [2]. As adherence is considered as a behavior, strategies that assess frequency or duration (e.g., using adherence diaries) [1] after the subject has been oriented on performing prescribed exercises cannot provide reliable insights into adherence behavior. A previous review highlighted that adherence diaries lack predictive validity for functional outcomes and that there is an urgent need to develop valid and reliable measures to assess home-prescribed exercise adherence [1].

Low back pain, which is recognized as the number one cause of global disability, had an overall point prevalence of 7.3% in 2015, implying that 540 million people worldwide were affected [3]. Low back pain is the leading chronic health problem in the world [4]. Current guidelines encourage active treatments for patients with Chronic Low Back Pain (CLBP) [5–7] since inactivity contributes negatively to recovery [7]. This gradual shift from exercise interventions administered in clinical settings to home exercise programs [8] encourages patients to change their lifestyles, engage in models of shared decision-making and save on costs. However, the assessment of the adherence to prescribed home exercises is the sine qua non for investigating the relationship between engagement, dose, and effectiveness. A systematic review [9] emphasized that the majority of the studies that assess the effects of exercise interventions did not investigate adherence to exercise. Long-term adherence to home exercise programs is important for patients with CLBP to maintain lasting benefits and reduce health costs [1], given the persistence of the condition. Considering such gap in the literature, Newman-Beinart et al. [10] developed the Exercise Adherence Rating Scale (EARS), which is a brief self-report measure comprised of three sections; the second section (B), with six items, is used to assess adherence behavior [11]. The original scale demonstrated acceptable outcomes in a population with CLBP [10].

Before a patient-reported outcome measure (PROM) is used to evaluate individuals from other countries and different cultures, it must be translated into the intended language and culturally adapted to the country in which it will be used [12, 13]. Moreover, before use in clinical or academic contexts, the measurement properties of the adapted version of the questionnaire should be established. COnsensus-based Standards for the selection of health Measurement Instruments (COSMIN) initiative recommends that instruments should be assessed regarding measurement properties in three main domains: reliability (the degree to which the measurement is free from measurement error), validity (the degree to which a PROM measures the construct(s) it purports to measure) and responsiveness (the ability of a PROM to detect change over time in the construct to be measured).

[14].

To our knowledge, there is no validated scale for assessing adherence to prescribed exercises in Brazilian Portuguese. Therefore, the objectives of this study were to translate and culturally adapt the original version of EARS to Brazilian Portuguese and test its measurement properties (construct validity, structural validity, internal consistency, reliability and responsiveness) in patients with non-specific CLBP.

## Methods

### Participants

One-hundred and eight patients between 18 and 60 years were enrolled in this study. They were recruited through medical referrals to physiotherapy outpatient service. Patients were contacted consecutively by phone, using their waiting list, and invited to participate in this study between August 2017 and February 2019. Patient eligibility was established using the following criteria: medical diagnosis of non-specific CLBP, pain in the last three months and/or pain on at least half of the days in the last six months [15], localized pain between the last thoracic vertebra and gluteal folds and people fluent in Brazilian Portuguese. Participants with a Mini-Mental State Examination (MMSE) score below cutoff values (better educated, score ≤ 23 and in the lower education, score ≤ 17) [16], illiterate people, with degenerative systemic diseases, neurological symptoms, lumbar stenosis, spondylolisthesis, history of spinal surgeries and pregnancy were excluded. Written informed consent was obtained from each patient, and their rights were protected. This study was approved by the Ethics Committee Board of the Centro de Saúde Escola Cuiabá from Ribeirão Preto School of Medicine – University of São Paulo (process number: 70955617.0.0000.5414) and.

### Procedure

All subjects participated in three sessions, including the following activities: Session (1) - baseline assessment (self-report questionnaires) and prescription of home exercise by a physiotherapist (motor control exercises); Session (2) - EARS administration after 1 week to investigate adherence behavior; Session (3) - retest of EARS and psychosocial reassessment – the retest was applied at a 1-week interval. Patients were contacted via telephone for responsiveness analysis, and the EARS, global perceived effect and numeric pain rating scales were reapplied three weeks after the first assessment. Figure 2 depicts a flow diagram illustrating the whole procedure.

### Instruments

Exercise Adherence Rating Scale (EARS) is a self-report measure developed by a group of United Kingdom researchers [10] that is composed of six items that directly assess adherence behavior (also called as Section B). The six items are summed and items with positive phrases are reversely scored; meaning items 1, 4 and 6. The six items are scored using an ordinal answer scale (0 = strongly agree to 4 = totally disagree), with higher scores indicating greater adherence (0 to 24). EARS was developed with two supporting optional sections: Section A and C. Section C has 10 items related to “reasons for adherence” or non-adherence (EARS-RA). Six additional questions, which allow open answers, were developed to obtain information about the exercise recommendations (Section A).

The Pain Self-Efficacy Questionnaire (PSEQ) [17] assesses confidence in the personal ability to perform well, despite the pain. It was translated to Brazilian Portuguese and validated [18]. The PSEQ has 10 items related to the tasks frequently reported as problematic by patients with chronic pain. The items are classified with an ordinal scale from 0 to 6; 0 = not confident and 6 = totally confident. A higher score reflects a stronger belief in self-efficacy (0 to 60).

The Fear-Avoidance Beliefs Questionnaire (FABQ) adapted and validated to Brazilian Portuguese [19], is composed of 16 items, with seven answer options each, from zero (completely disagree) to 6 (completely agree). The result should be obtained separately in each of the subscales. The work-related score ranges from 0 to 42 points, and the subscale related to physical activities ranges from 0 to 24 points.

The Pain Catastrophizing Scale (PCS) is a self-administered instrument developed to assess the degree of pain catastrophizing. It was adapted and validated for Brazilian Portuguese [20] and is composed of 13 items with answers ranging from 0 to 5 points. The patient must report the degree to which he/she recognizes any thought or feeling described by the item, and higher scores depict more severe pain catastrophizing. The instrument is subdivided into three subscales: amplification, rumination and helplessness.

The Hospital Anxiety and Depression Scale (HADS) is a self-administered scale used to identify anxiety and depression disorders in physically debilitated patients. This scale was translated and validated for Brazilian Portuguese [21]. It has two subscales, anxiety (HADS-A) and depression (HADS-D), with seven items in each domain. Each item has four response options ranging from 0 (“not at all”) to 3 (“most of the time”). The score for each subscale is up to 21 points and anxiety and/or depression is depicted by scores ≥8 points.

The Roland Morris Disability Questionnaire (RMDQ) assesses pain-related disability through statements related to activities of daily living. It is self-administered and has been adapted and validated for Brazilian Portuguese [22]. It has 24 items and the questionnaire score is calculated by adding the total number of questions marked with a “yes” answer. Thus, the score varies from 0 to 24 points, with 0 being the absence of disability and 24 being severe disability.

The Numeric Pain Rating Scale (NPRS) is a simple, easy-to-measure scale consisting of a sequence of integers, from 0 to 10; 0 represents “no pain” and 10 represents “worst possible pain”. The measurements have acceptable levels of reliability [23].

The Global Perceived Effect (GPE) is a Likert-type, 11-point scale (ranging from − 5 to + 5) that compares the patient’s current condition with his or her condition at the onset of symptoms. Positive and negative scores are assigned to patients who are better and worse, respectively [23].

### Measurement property studies

To make the interpretation of the results easier, all the measurement properties adopted, the methods, statistical analysis and results were described separately in different studies.

### Study 1 - cross-cultural adaptation of the EARS to Brazilian Portuguese and pre-testing

**Fig. 1:**
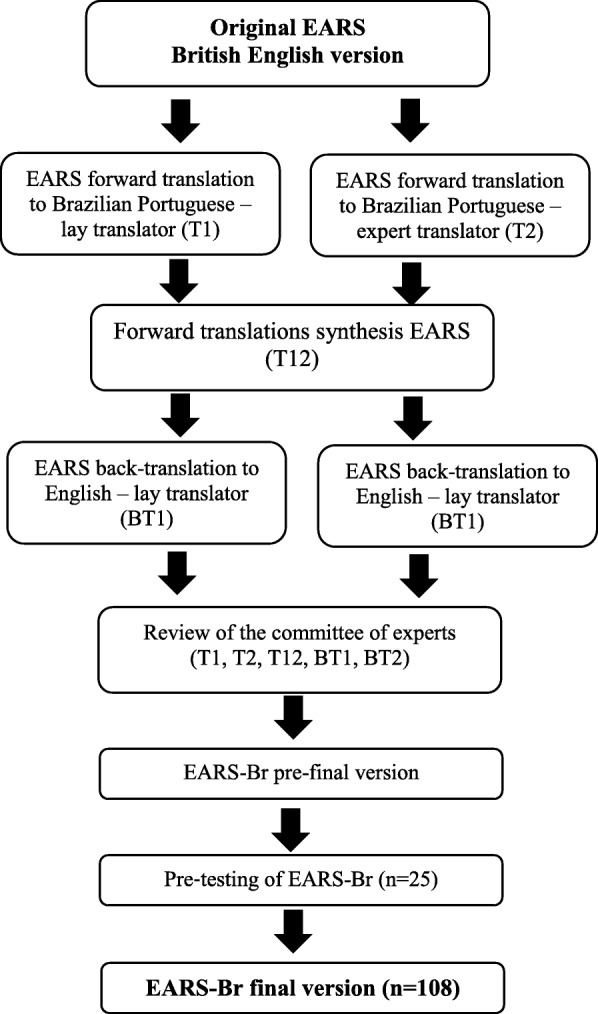
Flowchart of the process of cross-cultural adaptation to Brazilian Portuguese of the Exercise Adherence Rating Scale (EARS) in five stages: I) initial translation into Brazilian Portuguese – original version in British English was translated into Portuguese by two translators fluent in English and native speakers of Portuguese - a layman and an expert in health sciences, who worked independently; II) synthesis of translations – both translations were synthesized through consensus; III) back-translation into the original language – two translators fluent in Portuguese and native English speakers back-translated the synthetized version into English. They worked independently and both were blinded to the original version; IV) specialist committee – meeting with translators (*n* = 4), physiotherapists (*n* = 6) and PhDs and researchers with expertise in exercise (n = 4) to solve possible disagreements in translation, and create a pre-final version of EARS-Br and V) pre-testing phase – in which the pre-final version of the questionnaire was administered to patients with CLBP (*n* = 25) and assessed regarding comprehensibility of the instrument controlled through an open field form and cognitive interviews. Participants were encouraged to report their possible doubts, impressions of each item, response options, header items, instructions, and instrument layout

Initially, we requested the permission of the author of the original scale for the cross-cultural adaptation (E. L. Godfrey). The process followed a guideline commonly used in research [12, 13]. The cross-cultural adaptation process is detailed in Fig. 1.

### Study 2 - reliability and internal consistency

Eighty-three respondents of the final version of EARS-Br were asked to complete the questionnaire again after one week, to check for test-retest reliability. The one-week period was previously recommended [24]. For this stage, we considered only individuals with clinical stability and variations of less than 2 on the NPRS [25].

We also assessed measurement error through distribution-based methods: standard error of measurement (SEM) and minimally detectable change (MDC).

### Study 3- construct validity (structural validity included)

Construct validity can be defined as the degree to which the scores of an instrument are consistent with the hypotheses and it could be obtained by comparisons with other instruments [24]. To evaluate construct validity, we assessed structural validity and conducted hypothesis testing.

The structural validity estimates the degree to which the scores of a measuring instrument are adequate reflections of the dimensionality of the construct to be measured. Although the factor structure of EARS was previously described by exploratory factor analysis (EFA) [10], we adopted confirmatory factor analysis (CFA) [26] since the better approach was to confirm the factor structure.

For the construct validity - hypothesis testing, we ran correlations between the EARS-Br score and comparator instruments scores. Relationships between test scores and other measures intended to assess the same or similar constructs provide convergent validity, whereas relationships between measures of different constructs provide discriminant validity. It is assumed that discriminant validity is established by demonstrating that convergent correlations are higher than discriminant correlations [27]. For construct validity - hypothesis testing, we formulated a priori hypotheses based on previous publications [10], as follows:

H_1_) Mild/moderate, negative, and discriminant correlations between EARS-Br scores vs. FABQ, PCS, HADS, pain intensity (NPRS), and disability (RMDQ),

H_2_) Mild/moderate, positive, and discriminant correlation between EARS-Br scores vs. PSEQ,

H_3_) Moderate to strong, positive, and convergent correlation between EARS-Br vs. EARS-RA-Br,

H_4_) Higher correlations between EARS-Br vs. EARS-RA-Br than the other comparisons tested.

If 75% of the hypotheses are confirmed, construct validity is considered suitable [24].

### Study 4- responsiveness

The responsiveness refers to the ability of an instrument to detect change at two different time points, or the ability of an instrument to change relative to the change of a reference measure (external anchor) [24]. We assessed responsiveness using two construct approaches as defined by the COSMIN Study Design checklist for patient reported outcome measures [26]: (a) correlation between changes in scores and hypotheses testing; (b) comparison with other outcome measurement instruments.

For responsiveness based on the construct approach, we expected moderate to strong positive correlations between EARS change and GPE scores (H_5_) and moderate to strong negative correlations between EARS change and pain intensity scores (H_6_). We also checked for accuracy using the receiver operating characteristic curve (ROC curve) and minimally important change (MIC). The MIC is defined as the smallest change in score in the construct to be measured, which is perceived as important by patients, clinicians, or relevant others [28]. The reference measure adopted to assess MIC was the GPE (external anchor). Therefore, we raised hypotheses a priori: H_7_) moderate accuracy of EARS change score to detect who improved (increase in the score) on GPE and H_8_) moderate accuracy of EARS final score to detect who improved (reduction in the score) on GPE. A higher score on EARS-Br correlated with a higher improvement in GPE. Two units of change were deemed as an improvement on GPE [29].

### Statistical analysis

#### General statistics

Analysis of Variance (ANOVA) was performed to test for significant differences between subsamples of the different studies included in the project (*p* < 0.05). All analyses were performed using the SPSS statistical package for Windows and IBM SPSS, version 22.

#### Study 2 - reliability and internal consistency

Reliability was calculated using the intraclass correlation coefficient (ICC_2,1_, two-way random effect model). ICC values were classified as poor (< 0.40), moderate (0.40–0.75) and excellent (> 0.75) [30]. We calculated the SEM and MDC [31]. MDC is the smallest change that can be detected by the instrument beyond measurement error [24]. Both MDC and MIC (see below on study 4) should be higher than SEM [24].

The standard error of measurement (SEM) was analyzed using the following formula: SEM = SD x √(1 - ICC), in which SD = standard deviation.

The MDC is considered a distribution-based measure and was calculated as follows: MDC_95_ = 1.96 x √2 x SEM.

The internal consistency was analyzed using Cronbach’s α with acceptable results between 0.70 and 0.95 [24].

#### Study 3 – construct validity

To check for the structural validity of the EARS-Br scales, CFA was used. We analyzed the goodness of fit of three models: i) EARS-Br with a one-factor model with 6 items [8]; ii) One-factor EARS-RA-Br model with 10 items and iii) One-factor EARS-RA-Br with 9 items (item 8 was excluded). We investigated the factoriability of the dataset using an EFA approach assessing the following measures: Kaiser-Meyer-Olkin (KMO) with acceptable values ranging from 0.5 to 1 [32] and Bartlett’s test of sphericity, for which a cut-off below of 0.05 is recommended [33].

IBM SPSS AMOS (version 22) was used to run the CFA. As we identified a violation of multivariate normality, we run the analysis using a bootstrap maximum likelihood (ML) method (2000 resamples) [34]. Bollen–Stine gauges fit without normal theory limitations [35], and *p* > 0.05 suggests the acceptance of the null hypothesis of global fit (the model is correct).

Acceptability of fit was evaluated based on several indexes: root mean square error of approximation (RMSEA, recommended value below 0.08), comparative fit and goodness of fit indexes (CFI and GFI, recommended value close to 0.90), Expected Cross-Validation index and Consistent Akaike Information Criterion (ECVI and CAIC – lower values, best fit [36]), and CMIN/df (degrees of freedom) - should be less than 3 [36]. The magnitudes of factor loadings of 0.3 or greater [37] were considered suitable.

To assess for construct validity, hypothesis testing and spearman’s rho were used, and coefficients above 0.7 were classified as strong, those between 0.69 and 0.3 as moderate, and those below 0.29 as mild/weak [38].

#### Study 4 – responsiveness

We adopted three analyses to check for responsiveness: (a) correlation between change scores (construct approach, hypotheses testing - comparison with other outcome measurement instruments), (b) Determining the MIC for EARS-Br anchor-based responsiveness and (c) Determining the cut-off score for EARS-Br.

#### Correlation between change scores (construct validity - hypotheses testing)

We calculated the correlation between mean changes in scores for EARS vs. GPE and EARS vs. pain intensity using the Spearman rank correlation. The same classification for grading the magnitude of correlation was used, as described above [38].

#### Determining the MIC for EARS-Br

The MIC should be measured using an anchor-based approach in which an external anchor is adopted to run comparisons. We used GPE in the current study. We adopted the following metric to obtain the change in scores – EARS_MIC_: EARS final score (4th week) – EARS initial score (2nd week).

To calculate MIC, receiver operating characteristic (ROC) curves were plotted showing sensitivity and 1-specificity values and area under the curve (AUC) showing the probability of correctly discriminating between patients who improved (a change of at least 2 units as a criterion for improvement) and worsened/remained stable according to GPE (reference measure). The MIC for EARS was determined as the point of optimal cutoff in ROC curves related to greater sensitivity and specificity values [39], and higher than MDC [24].

#### Determining the cut-off score for EARS-Br

Beyond the MIC calculation, the EARS was used after the completion of the home exercise programs to assess adherence behavior retrospectively. A cut-off for the EARS score was also determined to guide the interpretability of EARS results. It was obtained by determining the minimum final EARS cut-off score of adherence behavior with a score of at least 2 units of improvement on GPE. The AUC classification used was: ROC> 0.9: high accuracy, 0.7 < ROC < 0.9: moderate accuracy, 0.5 < ROC < 0.7: low accuracy and ROC < 0.5: chance [39].

## Results

### Overall findings

**Fig. 2:**
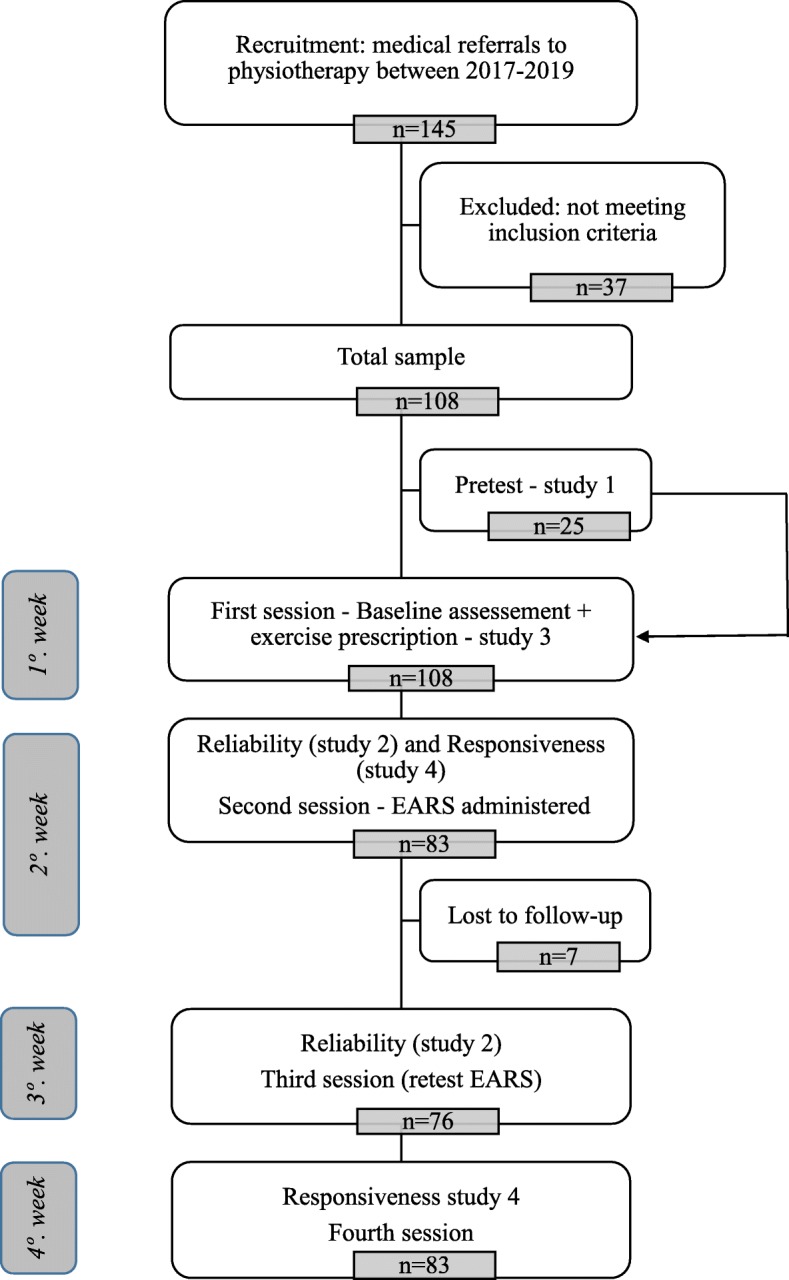
Flowchart showing sample distribution through EARS-Br validation study phases

Initially, 145 patients were invited to participate in this study and 37 were excluded because they did not meet the eligibility criteria. The final sample included 108 individuals. The pre-testing sample was comprised of 25 patients. For the test-retest reliability, we enrolled 76 participants (invited from the initial 108 participants) who had pain intensity changes less than 1 unit during the one week between baseline and test-retest assessments. Eighty-three patients with CLBP were assessed for responsiveness (Fig. 2). The clinical, educational and anthropometric data of the different subsamples of the studies are described in Table 1. A significant difference between subsamples considered in the distinct steps of the current study was observed only for the EARS-RA-Br score and the total score of the MMSE. However, for cognitive evaluation, all volunteers showed a cutoff value above the minimum for normal cognitive level [16].

**Table 1:** Description (mean and standard deviation: SD) of anthropometric, schooling and clinical/ psychosocial data of patients recruited in this study (*n* = 108)

Sample					
	Pre-testing sample Mean (SD)	Reliability sample Mean (SD)	Responsiveness sample Mean (SD)	Validity sample Mean (SD)	ANOVA (F_(3, 288)_, p)
Sample Size	25	76	83	108	
Age	46.33 (10.51)	46.84 (10.06)	46.71 (9.89)	46.62 (9.98)	0.01, *p* = 0.98
Weight	80.72 (19.44)	77.33 (14.93)	77.77 (14.84)	78.47 (16.01)	0.32, *p* = 0.72
Height	1.68 (0.10)	1.67 (0.08)	1.66 (0.08)	1.68 (0.09)	0.29 *p* = 0.74
Pain intensity	3.52 (2.27)	4.08 (2.91)	4.08 (2.87)	3.95 (2.74)	0.40, *p* = 0.66
Years lived with pain	7.80 (5.77)	6.06 (6.47)	6.06 (6.54)	6.74 (6.48)	0.71, *p* = 0.49
Female prevalence (%)	64% (*n* = 16)	67% (*n* = 52)	55% (*n* = 45)	57% (*n* = 61)	0.28, *p* = 0.75
Level of education^a^	1.6 (0.57)	1.69 (0.73)	1.6 (0.72)	1.6 (0.69)	0.19, *p* = 0.82
Mean score (SD)					
EARS	16.28 (6.32)	17.91 (6.71)	17.75 (6.62)	17.41 (6.55)	0.48, *p* = 0.61
EARS-RA	23.36 (7.39)	25.14 (6.41)	27.45 (6.67)	26.50 (7.03)	3.34, *p* = 0.03*
HADS anxiety (0–21)	9.64 (4.07)	7.34 (3.97)	8.39 (3.71)	9.10 (3.79)	0.32, p = 0.72
HADS depression (0–21)	6.60 (4.18)	5.36 (4.00)	6.66 (3.78)	6.64 (3.79)	0.00, *p* = 0.99
PESQ (0–60)	36.64 (17.57)	44.75 (13.58)	40.13 (15.27)	39.32 (15.81)	0.46, *p* = 0.62
PCS magnification (0–12)	5.40 (3.13)	4.68 (3.31)	4.76 (3.32)	4.91 (3.27)	0.07, *p* = 0.92
PCS rumination (0–16)	7.56 (3.90)	7.57 (3.44)	7.66 (3.51)	7.64 (3.58)	0.03, *p* = 0.97
PCS helplessness (0–24)	8.36 (6.05)	8.48 (5.96)	8.47 (5.98)	8.44 (5.96)	0.13, *p* = 0.87
FABQ phys (0–24)	12.56 (6.70)	10.73 (6.96)	14.30 (6.79)	13.89 (3.78)	0.63, *p* = 0.53
FABQ work (0–42)	27.8 (12.35)	21.77 (13.09)	25.04 (13.61)	25.68 (13.32)	0.40, p = 0.66
RMDQ (0–24)	12.8 (6.29)	11.69 (5.88)	13.42 (5.36)	13.27 (5.57)	0.11, *p* = 0.88

^a^Level of education: 1 = Incomplete/complete basic education; 2 = Incomplete/complete high school; 3 = Incomplete/complete higher education

EARS = Exercise Adherence Rating Scale; EARS-RA = Exercise Adherence Rating Scale, reasons for adherence; HADS = Hospital Anxiety and Depression Scale; PESQ = Pain Self-Efficacy Questionnaire; PCS = Pain Catastrophizing Scale; FABQ = Fear Avoidance Beliefs Questionnaire; RMDQ = Roland Morris Disability Questionnaire; MMSE = Mini-Mental State Examination

n = sample size. *p < 0.05

### Study 1 - cross-cultural adaptation of EARS-Br and pre-testing

During the meetings for cross-cultural translation and adaptation, there was a consensus on most of the questions among the members of the translation committee. However, the committee did not agree on one item of the EARS and two items of the EARS-RA. After a conversation with the author of the original version, the items were translated as below:

1. *“*I don’t get around to doing my exercises” - *Eu não consigo me organizar para fazer os meus exercícios* (the target meaning should be “cannot organize to do exercises”)
2. *“*I feel confident about doing my exercises” – *Eu sinto autoconfiança para fazer os meus exercícios* (the target meaning should be self-efficacy to exercise)
3. “I stop exercising when my pain is worse” - *Eu interrompo o exercício quando minha dor piora* (the target meaning should discontinue exercise when the pain gets worse, rather than to usually avoid exercising when the pain gets worse).

Moreover, the committee suggested the inclusion of descriptions for all possible response options and the authors of the original scale agreed with that adaptation. During the pre-testing, no volunteer reported any type of difficulty and/or suggestions for the EARS-Br.

The full questionnaire is available as a Supplementary File.

### Study 2 - reliability and internal consistency

Table 2 describes reliability, SEM, and MDC. For EARS-Br, the Cronbach’s α was acceptable (0.88). The ICC values for the EARS-Br scores were considered excellent (Table 2).

**Table 2:** Minimum and maximum score for EARS-Br items, mean score values, intraclass correlation coefficient (ICC), standard error of measurement (SEM) and minimally detectable change (MDC) of each question and total score of domains of the Brazilian Portuguese version of the Exercise Adherence Rating Scale (EARS-Br) (n = 108)

Items	ICC (95% CI) *n* = 83	SEM/ MDC n = 108	Mean Value EARS-Br (SD)	Minimum-maximum Value EARS-Br
1. I do my exercises as often as recommended	0.87 (0.80–0.92)	0.45/ 1.25	3.13 (SD = 1.25)	0–4
2. I forget to do my exercises	0.80 (0.69–0.87)	0.60/ 1.66	2.54 (SD = 1.34)	0–4
3. I do less exercise than recommended by my healthcare professional	0.92 (0.88–0.95)	0.47/ 1.30	2.83 (SD = 1.56)	0–4
4. I fit my exercises into my regular routine	0.86 (0.78–0.91)	0.49/ 1.36	3.42 (SD = 1.26)	0–4
5. I don’t get around to doing my exercises	0.91 (0.87–0.94)	0.41/ 1.13	3.25 (SD = 1.44)	0–4
6. I do most, or all, of my exercises	0.89 (0.83–0.93)	0.43/ 1.20	3.58 (SD = 1.30)	0–4
Total score	**0.91 (0.86–0.94)**	**1.97/5.45**	**18.75 (SD = 6.56)**	**0–24**

n = sample size; CI = confidence interval 95%; SD = Standard Deviation

SEM = SD√1 − ICC

MDC95 = 1·96 × SEM × √2

### Study 3 – construct validity

**Figs. 3A and 3B:**
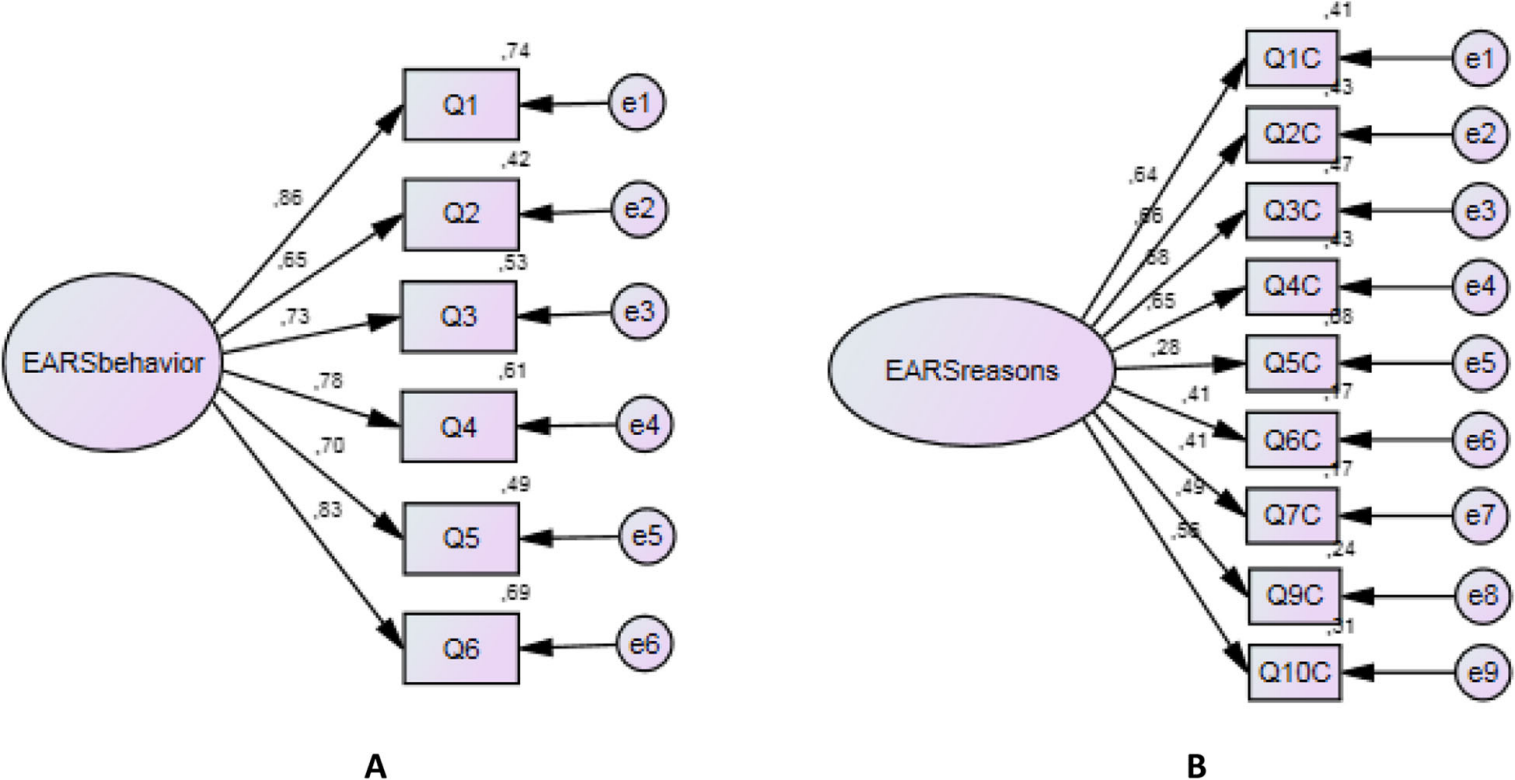
Path diagram showing factor structure of the EARS-Br (Fig. A) and EARS-Br reasons for adherence (Fig. B) describing the factor loadings for each item. Q = questions. e = error. EARSbehavior = EARS. EARSreasons = EARS reasons for adherence or EARS-RA

The EFA showed acceptable KMO values for EARS-Br and EARS-RA-Br (0.86 and 0.64), and Bartlett index (*p* < 0.001). Afterward, we investigated the fit of three different models as described in the statistical analysis. After the application of the bootstrap ML method, the Bollen–Stine *p*-value for the EARS-Br and EARS-RA-Br showed acceptable values. An acceptable fit was also observed for the EARS-Br with 6 items (Table 3). The factor loadings for both scales are depicted in Fig. 3. The EARS-RA-Br with 9 items also showed acceptable fit indexes (Table 3). Item 8 (I adjust the way I do my exercises to suit myself) was removed from the scale for “reasons for adherence” to exclusion improve the indices of fit (Table 3). Item 5 showed a poor factor loading (0.26), however, it was not excluded because it did not impair the overall fit of the scale (Table 3).

**Table 3:** Confirmatory factor analysis indices obtained for the Brazilian Portuguese version of the Exercise Adherence Rating Scale (EARS-Br) and for the EARS reasons for adherence (EARS-RA-Br) (*n* = 108)

	CMIN/df	CAIC	CFI	GFI	ECVI (90% CI)	RMSEA (90% CI)
**Models**	EARS-Br					
**EARS-Br – 6 items**^**#**^	1.89	70.14	0.97	0.93	0.38 (0.27–0.55)	0.08 (0.03–0.14)
	EARS-RA-Br					
**EARS-RA-Br 10 items**^**&**^	1.63	182.95	0.90	0.91	0.92 (0.77–1.13)	0.07 (0.03–0.11)
**EARS-RA-Br 9 items**^**&&**^	1.61	157.97	0.93	0.93	0.75 (0.62–0.94)	0.07 (0.02–0.12)

^#^One factor structure with 6 items relating to adherence behavior (6-items of section B) (Beinart et al., 2016)

^&^One factor structure with 10 questions relating to reasons for adherence (10-items of section C) of original EARS (Newman-Beinart et al., 2016)

^&&^One factor structure with 10 questions relating to reasons for adherence (10-items of section C) of original EARS (Newman-Beinart et al., 2016) with the exclusion of item 8

CMIN/df = χ^2^/df; CAIC = consistent Akaike information criterion; RMSEA = root-mean-square error of approximation; 90% CI = 90% confidence interval for RMSEA; CFI = comparative fit index; GFI = goodness of fit; ECVI = expected cross-validation index

n = sample size

Correlations between the EARS-Br scores and psychosocial scales are described in Table 4. We confirmed the hypotheses raised a priori (H_1_, H_3,_ H_4_), except for the correlation between PSEQ score and EARS-Br (H_2_) (Table 4).

**Table 4:** Correlations between EARS-Br vs. anxiety, depression, pain self-efficacy, fear of movement, disability, pain intensity, and reasons for adherence (*n* = 108)

Questionnaires and Domains (scores)	EARS-Br
HADS - Anxiety (0–21)	− 0.22*
HADS - Depression (0–21)	− 0.25*
PSEQ (0–60)	0.16
PCS- Helplessness (0–24)	− 0.22*
PCS- Magnification (0–12)	− 0.22*
PCS- Rumination (0–16)	− 0.27*
FABQ-Phys (0–24)	− 0.37**
FABQ-Work (0–42)	−0.21*
RMDQ (0–24)	−0.22*
NPRS (0–10)	−0.52**
EARS-RA-Br	0.86**

*p < 0.05, Spearman’s correlation

**p < 0.01, Spearman’s correlation

EARS = Exercise Adherence Rating Scale; HADS = Hospital Anxiety and Depression Scale; PESQ = Pain Self-Efficacy Questionnaire; PCS = Pain Catastrophizing Scale; FABQ = Fear Avoidance Beliefs Questionnaire; RMDQ = Roland Morris Disability Questionnaire; NPRS = Numerical Rating Scale; EARS-RA-Br = EARS reasons for adherence

n = sample size

### Study 4 – responsiveness

#### Correlation between change scores - construct approach, hypotheses testing

There was a moderate positive correlation in mean changes of scores between EARS-Br and GPE (r = 0.65, *p* < 0.001), and a moderate negative correlation between EARS-Br and pain intensity (NPRS) (r = − 0.58, p < 0.001). Hence, we confirmed our hypotheses H_5_ and H_6_.

#### Determining the MIC for EARS-Br

**Fig. 4:**
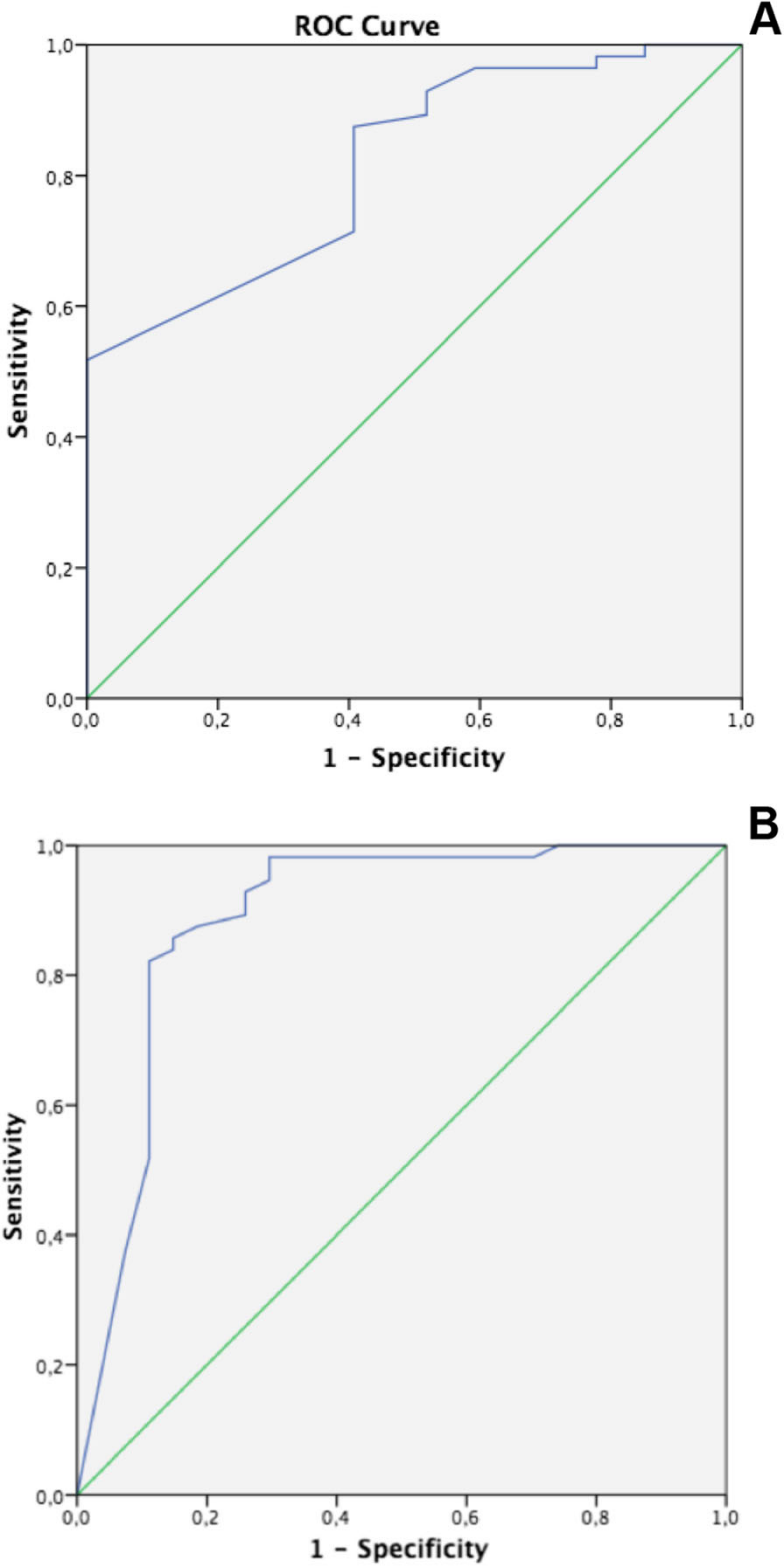
Receiver Operating Curves (ROC) describing sensitivity and specificity values for responsiveness analysis of the Brazilian version of the Exercise Adherence Rating Scale (EARS-Br). **A**: Minimally Important Change for EARS-Br considering as reference Global Perceived Effect (GPE), comparing EARS score longitudinally. **B**. Cut-off score for EARS-Br for the final assessment after the exercise program considering as reference Global Perceived Effect (GPE)

The responsiveness analysis showed moderate accuracy (AUC = 0.82) for a MIC of 5.5 (decrease) on EARS-Br, in distinguishing between patients that got worse or stable (*n* = 27) and those who improved (*n* = 57), considering the GPE as the reference measure (confirming H_7_). We showed a 93% sensitivity to detect those who reported worsening/stability and a 48% specificity to detect those who improved for the MIC of 5.5 (Table 5, Fig. 4a).

**Table 5:** Accuracy, cutoff, sensitivity and specificity values for the score of change (Minimally Important Change) and cut-off score for Exercise Adherence Rating Scale - Brazilian Portuguese (EARS-Br) in relation to Global Perceived Effect (GPE) (n = 83)

	Patients that improved	AUC (C.I. 95%)	Cutoff	Sensitivity	Specificity
**EARS-Br (change score) vs GPE**	56 (22.48, SD = 3.07)	0.82 (0.73–0.91)	5.5	0.93	0.48
**EARS-Br (cutoff score) vs GPE**	56 (22.48, SD = 3.07)	0.89 (0.79–0.98)	17.5	0.82	0.89

ROC> 0.9: high accuracy, 0.7 < ROC < 0.9: moderate accuracy, 0.5 < ROC < 0.7: low accuracy and ROC < 0.5: random outcome

AUC = Area Under the Curve

SD = standard deviation

GPE = Global Perceived Effect scale

#### Determining the cut-off score for EARS-Br

We also found a moderate accuracy (AUC = 0.89) for the cut-off of 17/24 on EARS-Br to distinguish between patients who improved (n = 57) and those who got worse or stable (n = 27) when considering GPE as the reference measure (confirming H_8_). We showed a sensitivity (ability to detect who improved on GPE) and specificity (ability to detect who got worse or stable) higher than 80% for the cut-off EARS score of 17 (Table 5, Fig. 4b).

## Discussion

This study carried out the cross-cultural adaptation of the EARS [8] for Brazilian Portuguese in patients with CLBP following international recommendations [13, 26]. EARS-Br showed excellent acceptability and comprehension during pre-testing and psychometric analyses. It also demonstrated acceptable reliability, internal consistency, construct and structural validity, and responsiveness. It is the first valid PROM available in Brazilian Portuguese that evaluates behavior adherence to prescribed exercises in patients with CLBP.

### Study 2 - reliability and internal consistency

The test-retest reliability of the EARS-Br scores was considered excellent for both EARS-Br scales, and our findings are supported by the results of the original version of the scale [10]. The SEM and MDC values for EARS-Br were 1.97 and 5.45, respectively. For the original EARS, such values were not described. The MDC obtained in our study showed that any MIC for the EARS-Br scores should be higher than 5.45 to surpass the measurement error.

Additionally, our findings showed an acceptable internal consistency (Cronbach’s α > 0.80) for EARS-Br, which is consistent with the internal consistency results reported by the original 6-item EARS (α = 0.81) [10]. We did not test the internal consistency of the EARS-RA-Br because the recommendation was against adding up its items to obtain a final score [10].

### Study 3 – construct validity

CFA confirmed the structure (structural validity) reported for the original EARS with 6 items, and we also checked for the structure of the EARS-RA. We showed an acceptable fit for that scale with 9 items. For EARS-RA-Br, two items showed factor loadings below 0.30. Despite the poor factor loading for both items, we decided to remove only the item that impaired the scale fit (item 8). That item was the only one that did not show a correlation with the EARS-Br total score as reported in the manuscript of the original version [10]. We cannot compare our results with the findings reported for the original EARS-RA, because it was not submitted to structural validity analysis. As recommended in the original manuscript, the items of EARS-RA should not be added up to obtain a total score. However, it is recommended that items are analyzed separately to determine which specific factors significantly influence adherence behavior.

For construct validity, we hypothesized a mild to moderate correlation for the psychosocial questionnaires administered (discriminant validity), and we observed that higher scores on fear-avoidance and pain intensity lowered the scores on EARS-Br. We also showed that higher scores for anxiety, depression, disability, fear-avoidance and pain catastrophizing lowered scores on EARS-Br. In agreement with our findings, a systematic review that identified barriers to adherence to treatment in physiotherapy outpatients showed that pain intensity, depression and anxiety were identified as barriers to adherence to exercise [40].

In this study, a correlation between pain self-efficacy and adherence behavior was not observed. Several studies have shown that poor self-efficacy could explain a patient’s low confidence in their ability to overcome obstacles to initiating, maintaining or resuming from relapses in exercises [41]. On the other hand, there is no question specifically related to exercise on PSEQ, since PSEQ is a questionnaire focused on pain self-efficacy and not exercise self-efficacy. This may explain our results since self-efficacy is a task-specific construct [42]. A new instrument has been described in literature and is available for specifically assessing self-efficacy for home prescribed exercises [43]. Future studies correlating exercise self-efficacy and adherence behavior (EARS) are therefore recommended.

We found a moderate and negative correlation between pain intensity and EARS-Br (discriminant validity). A greater intensity of pain correlated with a lower exercise adherence score. The original EARS study [10] also reported a moderate correlation between pain intensity and adherence. This may be related to the strong common patient belief that pain is a marker of tissue damage [44] and that exercise/movement may aggravate tissue damage and, consequently, pain. In a previous systematic review [40], pain intensity was also identified as a barrier to adherence, which is consistent with our findings. These results suggest that patients with higher levels of pain intensity may demonstrate worse adherence in clinical trials.

Ultimately, we found that correlations were higher between EARS-Br (adherence behavior) and EARS-RA-Br than between EARS-Br and the remaining constructs. Since both scales assess complementary aspects of exercise adherence, we consider these findings as parameters of discriminant validity [27].

### Study 4 – responsiveness

EARS is an instrument to be administered after the prescription of exercises and not as a tool to assess pre- and post-intervention change. Hence, a greater EARS score at the end of the exercise program correlated with better adherence to prescribed exercise protocols. However, EARS can be used to longitudinally monitor patient adherence to exercise, and it is important to define a minimum parameter of score fluctuation when following patients prescribed with home exercises. We controlled the change in EARS scores (after home exercise prescription) and its relationship with perceived improvement in two time points. Our findings showed that an acceptable fluctuation in EARS total scores should not exceed 5.5 (MIC). Therefore, any decrease in the total EARS score greater than 5.5 during the follow-up assessments should be interpreted as a meaningful decrease in adherence behavior to home exercises, and health professionals should intervene and identify the motivations for poor adherence. The MIC for EARS showed an excellent sensitivity for detecting patients who did not report improvement on GPE (93%), although it showed a low specificity for detecting those who improved (48%). This suggests that EARS scores may be better for evaluating the non-adherence behavior associated with a poor perception of improvement.

Additionally, we observed that the cut-off score of 17 distinguished between patients that perceived an improvement greater than 2 units on GPE. Our results suggest that the acceptable total score should be at least 17/24 considering the GPE score as a reference, and we recommend this as a guide for controlling adherence behavior. It was not possible to draw comparisons between the responsiveness outcomes of our study and the original scale, as we did not perform the required analysis.

### Limitations

Our study validated EARS-Br in a population with CLBP, hence the extrapolation of our results to other populations should be made with caution. It may also be valuable to investigate the validity of the EARS concurrently with an objective activity device, due to self-report bias when considering interventions that permit control for step counting (pedometers). However, there is no objective measure of adherence available for interventions used in physical therapy settings. Finally, we assessed for responsiveness during a short period of three weeks and using a small sample size. We suggest additional studies to investigate responsiveness during longer periods between assessments and using bigger sample sizes.

### Strength and clinical implications

The EARS-Br is the first validated tool in Brazilian-Portuguese that can assess adherence to prescribed home exercises in patients with CLBP. The scale showed acceptable measurement properties and can be used in clinical practice to follow-up on patients prescribed with home exercise programs. It can be adopted or used to monitor exercise adherence levels after hospital/outpatient discharge. A total EARS cut-off score of 17/24 could be used as a parameter of acceptable adherence behavior. Additionally, any decrease of 5.5 or more in the total EARS score could be adopted as a meaningful decrease in exercise adherence.

## Conclusion

The EARS scales were cross-culturally adapted for Brazilian Portuguese following international recommendations. EARS-Br is a reliable and valid instrument to assess adherence to prescribed home exercises in patients with CLBP. A final score of 17/24 on EARS after the prescription of home-exercise could be used as a cut-off for acceptable adherence behavior associated with improvement in patient outcomes.

## Supplementary information

**Additional file 1.** The full version Exercise Adherence Rating Scale in Brazilian Portuguese is available as Supplementary File

## Data Availability

The datasets used and/or analyzed during the current study are available from the corresponding author on reasonable request.

## Abbreviations

CLBP: Chronic low back pain
EARS: Exercise Adherence Rating Scale
PROM: Patient reported outcome measure
COSMIN: COnsensus-based Standards for the selection of health Measurement Instruments
MMSE: Mini-Mental State Examination
EARS-RA: Exercise Adherence Rating Scale - reasons for adherence or non-adherence
PSEQ: Pain Self-Efficacy Questionnaire
FABQ: Fear-Avoidance Beliefs Questionnaire
PCS: Pain Catastrophizing Scale
HADS: Hospital Anxiety and Depression Scale
HADS-A: Hospital Anxiety and Depression Scale - anxiety
HADS-D: Hospital Anxiety and Depression Scale - depression
RMDQ: Roland Morris Disability Questionnaire
NPRS: Numeric Pain Rating Scale
GPE: Global Perceived Effect
EFA: Exploratory Factor Analysis
CFA: Confirmatory actor analysis
ROC Curve: Receiver operating characteristic curve
MIC: Minimally Important Change
MDC: Minimally Detectable Change
ICC: Intraclass Correlation Coefficient
SEM: Standard Error of Measurement
KMO: Kaiser-Meyer-Olkin
ML: Maximum Likelihood
RMSEA: Root mean square error of approximation
CFI: Comparative fit
GFI: Goodness of fit indexes
ECVI: Expected Cross Validation Index
CAIC: Consistent Akaike Information Criterion
ROC: Receiver Operating Characteristic
AUC: Area Under the Curve
ANOVA: Analysis of Variance
