# Supplementary material for: The Brazilian Portuguese version of the Exercise Adherence Rating Scale (EARS-Br) showed acceptable reliability, validity and responsiveness in chronic low back pain
Source: BMC Musculoskelet Disord. 2020 May 12;21:294. doi: 10.1186/s12891-020-03308-z (PMC7218635; doi:10.1186/s12891-020-03308-z)
Supplement: Supplementary file 1 — Additional file 1. The full version Exercise Adherence Rating Scale in Brazilian Portuguese is available as Supplementary File [file 12891_2020_3308_MOESM1_ESM.docx]

**Escala de Avaliação de Adesão ao Exercício (EARS-Br)**

**Seção A: Questionário sobre Exercícios Prescritos**

Os profissionais de saúde normalmente recomendam que pessoas com condições crônicas de saúde façam exercícios e/ou atividades para melhorar sua qualidade de vida e lidar melhor com sua condição. As pessoas geralmente encontram sua própria maneira de fazer seus exercícios/atividades. Nós gostaríamos que você nos contasse como você costuma fazer seus exercícios/atividades.

**Por favor, marque todas os quadrados que se aplicam a você.**

**1) Qual exercício/ atividade você foi solicitado a fazer?**

          Sessões de exercícios individuais com um profissional de saúde

          Sessões de exercícios em grupo

          Exercícios individualizados para fazer em casa, conforme recomendado por um profissional de saúde

          Exercícios regulares em geral

          Caminhar

          Permanecer ativo em sua vida diária

          Outros_______________________________________________________________

**2) Com que frequência você foi convidado a fazer esses exercícios e/ ou atividades?**

          todo dia

          4 a 6 dias por semana

          2 a 3 dias por semana

          1 dia por semana

          Menos do que isso

          Outro________________________________________________________________

**3) Por quanto tempo você foi convidado a continuar fazendo esses exercícios e/ ou atividades?**

         Contínuo

         Por uma duração fixa (por favor, especifique-se) ______________________________

         Outro (por favor, indique) _________________________________________________

4**) Com que frequência você está fazendo esses exercícios e/ ou atividades?**

          todo dia

          4 a 6 dias por semana

          2 a 3 dias por semana

          1 dia por semana

          De modo nenhum

**5) Se você parou de fazer seus exercícios / atividades, quando você parou e por quê?**

**6) Em suas próprias palavras, por favor, você pode nos dizer por que você fez ou não, seus exercícios?**

**Seção B: Comportamento de adesão**

Para cada uma das 6 frases abaixo, por favor marque com um “X” no quadrado que melhor descreva a maneira como você faz seus exercícios/atividades recomendados. Ao pensar em suas respostas, por favor considere quaisquer exercícios/atividades que você foi convidado a fazer como parte do seu tratamento.

1. **Eu faço os meus exercícios de acordo com a frequência recomendada**

| Concordo totalmente | Concordo parcialmente | Nem discordo, nem concordo | Discordo parcialmente | Discordo totalmente |
| --- | --- | --- | --- | --- |
| ***0*** | ***1*** | ***2*** | ***3*** | ***4*** |
|  |  |  |  |  |

1. **Eu esqueço de fazer os meus exercícios**

| Concordo totalmente | Concordo parcialmente | Nem discordo, nem concordo | Discordo parcialmente | Discordo totalmente |
| --- | --- | --- | --- | --- |
| ***0*** | ***1*** | ***2*** | ***3*** | ***4*** |
|  |  |  |  |  |

1. **Eu faço menos exercício do que o recomendado pelo meu professional de saúde**

| Concordo totalmente | Concordo parcialmente | Nem discordo, nem concordo | Discordo parcialmente | Discordo totalmente |
| --- | --- | --- | --- | --- |
| ***0*** | ***1*** | ***2*** | ***3*** | ***4*** |
|  |  |  |  |  |

1. **Eu encaixo os meus exercícios na minha rotina**

| Concordo totalmente | Concordo parcialmente | Nem discordo, nem concordo | Discordo parcialmente | Discordo totalmente |
| --- | --- | --- | --- | --- |
| ***0*** | ***1*** | ***2*** | ***3*** | ***4*** |
|  |  |  |  |  |

1. **Eu não consigo me organizar para fazer os meus exercícios**

| Concordo totalmente | Concordo parcialmente | Nem discordo, nem concordo | Discordo parcialmente | Discordo totalmente |
| --- | --- | --- | --- | --- |
| ***0*** | ***1*** | ***2*** | ***3*** | ***4*** |
|  |  |  |  |  |

1. **Eu faço a maioria, ou todos, os meus exercícios**

| Concordo totalmente | Concordo parcialmente | Nem discordo, nem concordo | Discordo parcialmente | Discordo totalmente |
| --- | --- | --- | --- | --- |
| ***0*** | ***1*** | ***2*** | ***3*** | ***4*** |
|  |  |  |  |  |

**Seção C: Razões de adesão ao exercício**

Para cada uma das 9 frases abaixo, por favor marque com um “X” no quadrado que melhor descreva o motivo pelo qual você faz ou não seus exercícios/atividades recomendados.

1. **Eu não tenho tempo para fazer os meus exercícios**

| Concordo totalmente | Concordo parcialmente | Nem discordo, nem concordo | Discordo parcialmente | Discordo totalmente |
| --- | --- | --- | --- | --- |
| ***0*** | ***1*** | ***2*** | ***3*** | ***4*** |
|  |  |  |  |  |

1. **Outros compromissos impedem que eu faça os meus exercícios**

| Concordo totalmente | Concordo parcialmente | Nem discordo, nem concordo | Discordo parcialmente | Discordo totalmente |
| --- | --- | --- | --- | --- |
| ***0*** | ***1*** | ***2*** | ***3*** | ***4*** |
|  |  |  |  |  |

1. **Eu não faço os meus exercícios quando estou cansado(a)**

| Concordo totalmente | Concordo parcialmente | Nem discordo, nem concordo | Discordo parcialmente | Discordo totalmente |
| --- | --- | --- | --- | --- |
| ***0*** | ***1*** | ***2*** | ***3*** | ***4*** |
|  |  |  |  |  |

1. **Eu sinto autoconfiança para fazer os meus exercícios**

| Concordo totalmente | Concordo parcialmente | Nem discordo, nem concordo | Discordo parcialmente | Discordo totalmente |
| --- | --- | --- | --- | --- |
| ***0*** | ***1*** | ***2*** | ***3*** | ***4*** |
|  |  |  |  |  |

1. **Minha família e amigos me motivam a fazer os meus exercícios**

| Concordo totalmente | Concordo parcialmente | Nem discordo, nem concordo | Discordo parcialmente | Discordo totalmente |
| --- | --- | --- | --- | --- |
| ***0*** | ***1*** | ***2*** | ***3*** | ***4*** |
|  |  |  |  |  |

1. **Eu faço os meus exercícios para melhorar a minha saúde**

| Concordo totalmente | Concordo parcialmente | Nem discordo, nem concordo | Discordo parcialmente | Discordo totalmente |
| --- | --- | --- | --- | --- |
| ***0*** | ***1*** | ***2*** | ***3*** | ***4*** |
|  |  |  |  |  |

1. **Eu faço os meus exercícios porque gosto deles**

| Concordo totalmente | Concordo parcialmente | Nem discordo, nem concordo | Discordo parcialmente | Discordo totalmente |
| --- | --- | --- | --- | --- |
| ***0*** | ***1*** | ***2*** | ***3*** | ***4*** |
|  |  |  |  |  |

1. **Eu interrompo o exercício quando minha dor piora**

| Concordo totalmente | Concordo parcialmente | Nem discordo, nem concordo | Discordo parcialmente | Discordo totalmente |
| --- | --- | --- | --- | --- |
| ***0*** | ***1*** | ***2*** | ***3*** | ***4*** |
|  |  |  |  |  |

1. **Eu não tenho certeza de como fazer os meus exercícios**

| Concordo totalmente | Concordo parcialmente | Nem discordo, nem concordo | Discordo parcialmente | Discordo totalmente |
| --- | --- | --- | --- | --- |
| ***0*** | ***1*** | ***2*** | ***3*** | ***4*** |
|  |  |  |  |  |

**Obtendo o escore da Escala de Avaliação de Adesão ao Exercício (EARS-Br)**

Esta informação de pontuação está relacionada aos 6 itens da EARS-Br (Seção B). A EARS-Br é pontuada em uma escala Likert de 5 pontos (0 - concorda totalmente a 4 - discorda totalmente). Os itens 1, 4 e 6 são pontuados inversamente, resultando em uma pontuação possível de 0 a 24 pontos. Um escore mais alto indica maior adesão.

**Adaptando a Escala de Avaliação de Adesão ao Exercício (EARS-Br)**

O Questionário sobre Exercícios Prescritos pode ser adaptado para atender às necessidades individuais (Seção A). O questionário EARS-Br de 6 itens validado não pode ser adaptado, pois isso tornaria o questionário inválido (Seção B). O questionário de 9 itens (razões de adesão ao exercício) consiste em itens relacionados às razões pelas quais um indivíduo pode ou não aderir ao exercício domiciliar prescrito (Seção C). Os itens 4, 5, 6 e 7 requerem pontuação inversa para que uma pontuação maior indique melhor adesão. Esses 9 itens podem ser usados ​​como itens únicos que podem ser adicionados ou adaptados para atender às necessidades individuais.
